# Optimal interval to surgery after chemoradiotherapy in rectal cancer: A protocol for systematic review and network meta-analysis

**DOI:** 10.1097/MD.0000000000017669

**Published:** 2019-11-11

**Authors:** Ya Jing Chen, Zhen-Jie Zhao, Bang Wei Wang, Guang Zhuang Jing, Hai-Kun Ma, Xuemei Han, Jiancheng Wang, Zhen-Jie Zhao

**Affiliations:** a Department of Social Medicine and Health Management, School of Public Health, Lanzhou University; b General Surgery Department 2, The First Hospital of Lanzhou University; c The First Clinical Medical College, Lanzhou University; d Gansu Provincial Hospital; e Hospital Management Research Center, Lanzhou University, Lanzhou, Gansu, China.

**Keywords:** chemoradiotherapy, meta-analysis, optimal interval to surgery, protocol, rectal cancer

## Abstract

**Background:**

Rectal cancer is the second leading cause of cancer-related death in the Western world. Preoperative neoadjuvant chemoradiotherapy (nCRT) has been widely performed in the treatment of rectal cancer patients. However, there is no consensus on the length of waiting interval between the end of preoperative nCRT and surgery. Present network meta-analysis (NMA) aims to compare the differences of effect between all available interval to surgery after nCRT in rectal cancer in improving overall survival, disease-free survival and pathologic complete response (pCR) rate, and to rate the certainty of evidence from present NMA.

**Method:**

We will systematically search PubMed, EMBASE, Chinese Biomedical Literature Database, and Cochrane Central Register of Controlled Trials (CENTRAL) databases to identify studies assessing the interval to surgery after CRT in rectal cancer. We will conduct this systematic review and meta-analysis using Bayesian method and report the full-text according to Preferred Reporting Items for Systematic Review and Meta-Analysis (PRISMA) Extension Vision statement (PRISMA-NMA). We will assess the risk of bias of individual study using the Newcastle–Ottawa Scale and Cochrane Handbook V.5.1.0. We will also use the advance of GRADE to rate the certainty of NMA. Data will be analyzed by using R software V.3.4.1.

**Results:**

The results of this study will be published in a peer-reviewed journal.

**Conclusion:**

To the best of our knowledge, this systematic review and NMA will first use both direct and indirect evidence to compare the differences of all available interval to surgery after CRT in rectal cancer. This is a protocol of systematic review and meta-analysis, so the ethical approval and patient consent are not required.

## Abstract 2

Supplemental Digital Content is available in the text

## 1 Introduction

Rectal cancer is among the most common cancers in the Western world and the second leading cause of cancer-related death.^[1,2]^ Treatment includes surgery, radiation and chemotherapy. Total mesorectal excision (TME) combined with preoperative nCRT in rectal cancer has result in significant improvement of tumor regression, lower rates of local recurrence, and leads to downsizing of the tumor.^[3–7]^ TME as the gold standard surgical procedure for rectal cancer, local recurrence rates have dropped to 5% to 10%.^[8]^ Swedish Rectal Cancer Trial results showed that short-course preoperative radiotherapy lower rates of local recurrence by more than 50%.^[9]^ Previously conducted studies^[3,7,10–12]^ have been demonstrated that preoperative nCRT and radiation therapy improve local control in patients with resectable rectal cancer. Ideally, surgery should take place at the time of optimal response to preoperative nCRT as the effects of the latter are time dependent.^[13]^

Although preoperative nCRT has been widely performed in the treatment of rectal cancer patients, the optimal timing for rectal cancer surgery after preoperative therapy still remains equivocal. Most surgeons tend to perform operations on the basis of a 6-week window after completion of nCRT for rectal cancer. Recently conducted studies demonstrated that pathological complete response (ypCR) was improved and tumor downstaging was significantly increased in patient who underwent surgery 6- to 8-week after completion of preoperative nCRT.^[14–19]^ One meta-analysis also showed that pre-operative nCRT followed by rectal surgery after a waiting interval longer than 6 to 8 weeks increases pCR.^[20]^ There is growing evidence that a prolongation of the treatment interval might improve overall survival (OS).^[17]^ However, other studies^[21,22]^ indicated that a waiting interval beyond 8 weeks is not advantageous and a longer than recommended interval between end of radiotherapy and surgery appeared to be associated with increased postoperative mortality. Evidence also suggested that a waiting interval beyond 8 weeks might increase fibrosis around the TME plane, potentially lead to intra-operative technical difficulties, and higher surgical morbidity.^[13]^

Network meta-analysis (NMA) has become increasingly popular to evaluate healthcare interventions, since it allows for estimation of the relative effectiveness among all interventions and rank ordering of the interventions even is head-to-head comparisons are lacking.^[23,24,25]^ We will conduct a comprehensive NMA aims to evaluate which would be the optimal time to operate by compared pCR, OS rate, disease-free survival rate (DFS), operative time, incidence risk of recurrence with five year, postoperative complications and quality of life in patients with rectal cancer. Meanwhile, GRADE approach will be used to rate the confidence or certainty of evidence from NMA, which could reflect the extent to which confidence in an estimate of the effect.^[26]^

## 2 Method

Our study protocol has been registered on the international prospective register of a systematic review (PROSPERO). The registration number was CRD42019137323. The systematic review protocol was planned and performed adherence to Preferred Reporting Items for Systematic Review and Meta-Analysis (PRISMA) Extension Vision statement (PRISMA-NMA).^[27]^

### 2.1 Eligibility criteria

#### 2.1.1 Types of study

We will include randomized controlled trails and observational comparative studies that compared outcomes between patients in whom surgery for rectal cancer was performed at different time intervention after chemoradiotherapy (CRT) delivered over “short interval” or “long interval”. We will include studies reported in any language.

#### 2.1.2 Types of patients

We will include studies involving participants with rectal cancer confirmed by biopsy, who receive preoperative CRT and undergo operation for rectal cancer.

#### 2.1.3 Types of interventions

We will include studies that comparing the efficacy and safety after neoadjuvant radiotherapy or chemoradiation followed by delayed surgery for the management of rectal cancer. The waiting intervals between the end of preoperative CRT and surgery reported in the included studies were not limited.

#### 2.1.4 Type of outcomes

The primary outcomes are pathologic complete response (pCR) rate, OS rate, DFS rate. The secondary outcome includes operative time, incidence risk of recurrence with 5 years, postoperative complications, and quality of life. Pathological complete response (pCR) was defined as the complete absence of tumor cells in the resected specimen and lymph nodes (ypT0N0),^[28]^ or no intact cancer cells found in the resected specimen regardless of the presence of mucin lakes,^[21]^ operative time was defined as time from skin incision to skin closure, length of hospital stay was defined as time from the index operation to discharge and postoperative pain was defined as visual analog scale (VAS) immediately after and during 1 week of the operation, recurrence was defined as clinical or radiologic recurrence of rectal cancer, complications was defined as any complications requiring further procedures in the theatre during the same surgical admission. Studies reporting on at least one related outcome will be included.

### 2.2 Data source

Two review authors will independently search the PubMed, EMBASE, Cochrane Central Register of Controlled Trials (CENTRAL) databases, and Chinese Biomedical Literature Database from their inception to April 2019 using the following key words: tim∗or Tim∗ or “interval” or “Delay∗” or “delay∗” or “interval∗” or “Intercal∗” and “rectal cancer” or “rectal carcinoma” or “rectal adenocarcinoma” or “rectal neoplasms”. A detailed search strategy can be found in Supplemental Digital Content (Appendix 1).

### 2.3 Study selection and data extraction

Literature search records will be imported into ENDNOTE X6 literature management software. Two independent interviewers will screen the title and abstracts of each citation retrieved according to eligibility criteria. Thus, full-text versions of all potentially relevant studies will be obtained and reviewed to ensure eligibility. We will create a study flow diagram to map out the number of records identified, included and excluded.^[29]^

We will use a standard data extraction form with detailed written instructions which will be created using Microsoft Excel 2013(Microsoft, Redmond, Washington, USA, www.microsoft.com) to collect data of interest. The information will include name of first author, year of publication, country in which the study was conducted, sample size, interventions, and outcomes. Study selection and data extraction will be performed by one reviewer, and the third reviewer to check. Any conflicts will be resolved by discussion or consultation of a third author.

When some trials report median rather mean, and range or interquartile range rather than SD (standard deviation), in which case the mean and SD will be estimated.^[30]^

### 2.4 Risk of bias of individual studies

Two reviewers will independently assess the risk of bias of each study using the Cochrane Handbook V.5.1.0 for systematic reviews of intervention to assess the quality of included RCTs,^[31]^ which focusing on biases related to 6 key domains: random sequence generation, allocation concealment, blinding of all participants, including patients, personnel and outcome assessors, incomplete outcome data, selective reporting, and other source of bias. Each domain will receive a judgment on the risk of bias (high, low and unclear). We will use the Newcastle-Ottawa scale (NOS) and Jadad score to assess the quality of the observational comparative studies. The NOS assessed for potential selection bias, the comparability of cased and controls or cohorts, and the ascertainment of outcome (case-control studies) or exposure (cohort studies). Points (also termed “stars”) are awarded and summed. Studies with ≥5 stars were considered high quality and were included into the study, and <4 stars were considered low quality and were excluded from the study. The Jadad scale is 5-point scale in which a score of 2 represents poor quality evidence and a score of 3 represents high quality evidence. Therefore, studies with a score of 3 to 5 were considered to be of high methodological quality. Any disagreement between the 2 reviewers will be resolved by discussion with a third review.

### 2.5 Geometry of the network

A network plot will be drawn using R software V.3.4.1. Nodes will be used to represent different interventions and edges to represent the head-to-head comparisons between interventions. The size of nodes is proportional to the number of studies evaluating a test, and thickness of the lines between the nodes is proportional to the number of direct comparisons between tests.^[32]^

### 2.6 Statistical analysis

#### 2.6.1 Pairwise meta-analyses

We will perform pairwise meta-analyses using random-effects model by R software V.3.4.1. We will use risk ratios (RR) with 95% confidence interval (95%) to measure dichotomous outcomes (including OS, DFS, recurrence, complications, pCR, downstaging rate, ypTNM stage) and the mean difference (MD) with 95% CI will be presented for continuous data (operative time and quality of life). The potential heterogeneity across head-to-head trials will be assessed by *I*^2^ statistics. If the *I*^2^ is ≦50%, it suggests that there is negligible statistical heterogeneity, and the fixed effects mode will be used for meta-analysis. If the *I*^2^ is >50%, we will explore sources of heterogeneity by subgroup analysis and meta-regression. If there is no clinical heterogeneity, the random effects model will be used to perform meta-analyses. We will use Begg and Egger funnel plot method through STATA V.12.0 software (Stata Corporation, CollegeStation, Texas) to examine publication bias when at least included 10 studies for on related outcome.^[33]^

#### 2.6.2 Network meta-analyses

A Bayesian random-effects NMA will be performed using package ‘*gemtc’* version 0.8–2 package of R-3.4.0 software.^[34]^ Its *mtc.run* function will be used to generate samples using the Markov chain Monte Carlo sampler, with four Markov chains run concurrently. We will set 5000 simulations for each chain as the “burn-in” period. Posterior summaries will be then based on 50,000 subsequent simulations. Convergence of models will be assessed using Brooks-Gelman-Rubin plots. We will use the node-splitting approach to obtain direct and indirect estimates, and tested the inconsistency for each comparison.^[35,36]^ We will use the surface under the cumulative ranking area (SUCRA) to rank the different time interventions. SUCRA values ranged from 0% to 100%, with higher values indicating a more effective intervention.^[37]^ The comparison-adjusted funnel plots will be conducted to assess the effects of the sample size on the results. A network plot will be drawn to describe and present the geometry of the intervention network of comparisons across studies to ensure if a NMA is feasible. We will exclude the trials that are not connected by interventions. All the result figures will be generated using R software V.3.4.

### 2.7 Subgroup analyses

We will perform subgroup analysis for RCTs. Sensitivity analysis will be performed excluding 1 study at a time, including studies with high overall risks of bias and studies that contained a proportion of patients undergoing neoadjuvant short-course radiotherapy (nSCRT) or intensification of the chemotherapy component to nCRT. The network meta-regression analyses will be conducted for all variables in the subgroup analyses to explain the between-trail heterogeneity observed.

### 2.8 Quality of evidence

We will use the Grading of Recommendations Assessment, Development and Evaluation^[38]^ (GRADE) to assess the quality of evidence for the primary outcomes, according to the comprehensive result of factors (risk of bias, inconsistency, indirectness, imprecision, publication bias) that influenced evidence quality which grades 4 levels: High level, moderate level, low level, and very low level.

## Acknowledgments

We thank all members of our study team for their whole-hearted cooperation and the original authors of the included studies for their wonderful work. We also thank Evidence-Based Medicine Center of Lanzhou University for methodological support.

## Author contributions

**Conceptualization:** Yajing Chen, Zhenjie Zhao.

**Investigation:** Yajing Chen, Bangwei Wang, Guangzhuang Jing, Haikun Ma, Xuemei Han.

**Methodology:** Yajing Chen.

**Project administration:** Yajing Chen, Bangwei Wang, Guangzhuang Jing, Haikun Ma.

**Supervision:** Jiancheng Wang, Zhenjie Zhao.

**Writing – original draft:** Yajing Chen, Zhenjie Zhao

**Writing – review & editing:** Yajing Chen, Zhenjie Zhao, Bangwei Wang, Guangzhuang Jing, Haikun Ma, Xuemei Han, Jiancheng Wang.

## Supplementary Material

Supplemental Digital Content
