# Supplementary material for: Optimal interval to surgery after chemoradiotherapy in rectal cancer: A protocol for systematic review and network meta-analysis
Source: Medicine (Baltimore). 2019 Nov 11;98(45):e17669. doi: 10.1097/MD.0000000000017669 (PMC6855568; doi:10.1097/MD.0000000000017669)
Supplement: Supplemental Digital Content [file medi-98-e17669-s001.doc]

| Box1 Sample search strategy for EMBASE |
| --- |
| #1‘neoplasms’/exp OR‘cancer*’OR‘tumor*’OR‘tumour*’OR‘carcinoma*’OR‘adenocarcinoma*’ OR ‘neoplas*’  #2‘rectum’/exp OR ‘rectum’ OR ‘ rectal’ OR ‘colorectal’ OR ‘recti’ OR ‘retrorectal’ OR ‘pararectal’  #3 #1 AND #2  #4‘radiotherapy’/exp OR ‘radiotherapy’ OR ‘irradiation’ OR  ‘chemotherapy’ OR ‘chemoradiation’ OR ‘chemoradiotherapy’/exp  OR ‘chemoradiotherapy’ OR ‘radiochemotherapy’ OR ‘combined modality’ OR ‘multimodal’ OR ‘neoadjuvant’ OR‘neoadjuvant’OR ‘surgery’OR‘anterior resection’ OR ‘total mesorectal excision’  #5‘Time’ /exp OR ‘tim*’ OR ‘Tim*’ OR ‘interval’ OR ‘Delay*’OR ‘delay*’ OR ‘interval*’ OR ‘Intercal*’  #6 #3 AND #4 AND #5 |
